# Supplementary figures and images for: The BDNF Val68Met polymorphism causes a sex specific alcohol preference over social interaction and also acute tolerance to the anxiolytic effects of alcohol, a phenotype driven by malfunction of BDNF in the ventral hippocampus of male mice
Source: Psychopharmacology (Berl). 2023 Jan 9;240(2):303–17. doi: 10.1007/s00213-022-06305-3 (PMC9879818; doi:10.1007/s00213-022-06305-3)

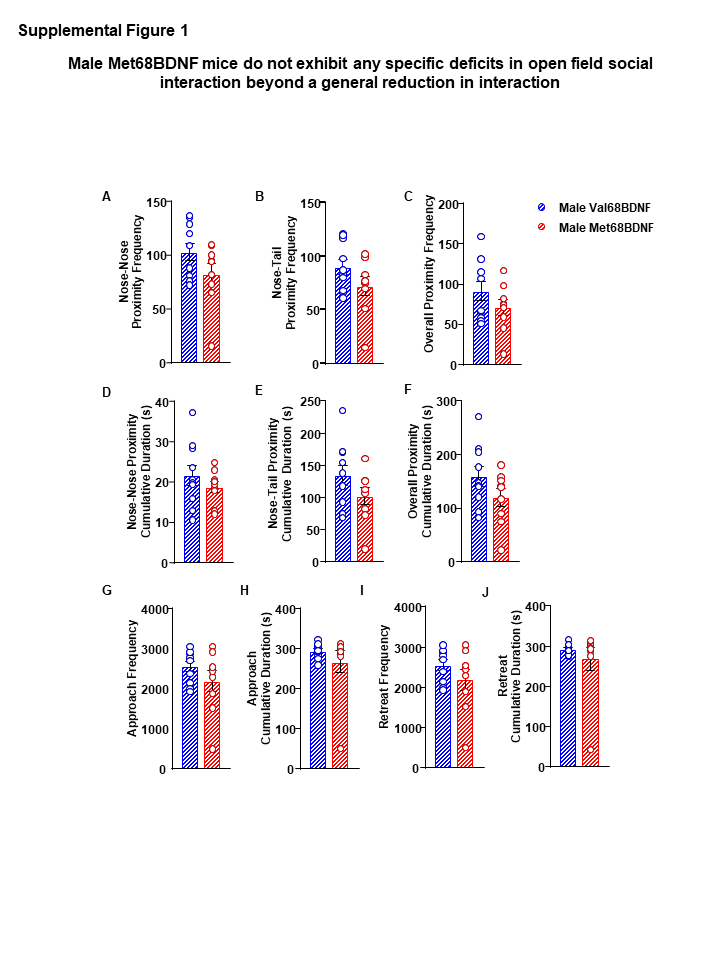

Supplement: Supplementary file 1 — Male Met68BDNF mice do not exhibit any specific deficits in open field social interaction beyond a general reduction in interaction (A) Male Val68BDNF and Met68BDNF mice came within 5 cm of their interaction partner (nose-to-nose) a similar number of times in the open field social interaction test. (B) Male Val68BDNF and Met68BDNF mice came within 5 cm of their interaction partner (nose-to-tail) a similar number of times in the open field social interaction test. (C) Male Val68BDNF and Met68BDNF mice came within 5 cm of their interaction partner a similar number of times in the open field social interaction test overall. (D) Male Val68BDNF and Met68BDNF mice spent a similar amount of time within 5 cm of their interaction partner (nose-to-nose) in the open field social interaction test. (E) Male Val68BDNF and Met68BDNF mice spent a similar amount of time within 5 cm of their interaction partner (nose-to-tail) in the open field social interaction test. (F) Male Val68BDNF and Met68BDNF mice spent a similar amount of time within 5 cm of their interaction partner in the open field social interaction test overall. (G) Male Val68BDNF and Met68BDNF mice approached their interaction partner a similar number of times in the open field social interaction test. (H) Male Val68BDNF and Met68BDNF mice spent a similar amount of time approaching their interaction partner in the open field social interaction test. (I) Male Val68BDNF and Met68BDNF mice retreated from their interaction partner a similar number of times in the open field social interaction test. (J) Male Val68BDNF and Met68BDNF mice spent a similar amount of time retreating from their interaction partner in the open field social interaction test. Data are represented as mean ± SEM. Val68BDNF: n = 10, Met68BDNF: n = 9. (PNG 70 kb) [file 213_2022_6305_Fig6_ESM.png]

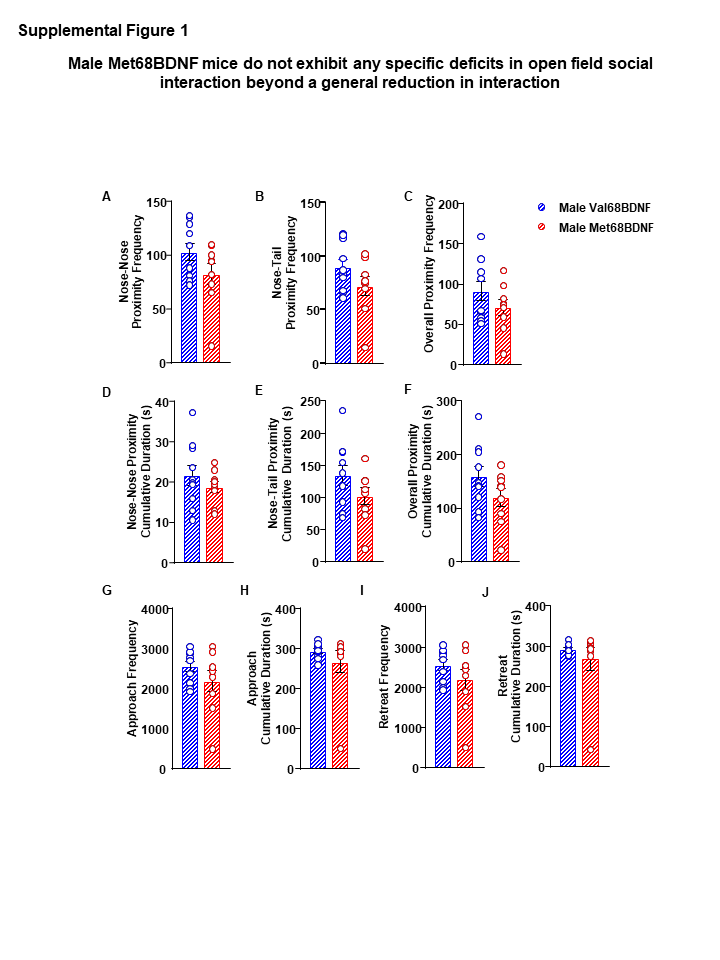

Supplement: Supplementary file 2 — High Resolution (TIF 123 kb) [file 213_2022_6305_MOESM1_ESM.tif]

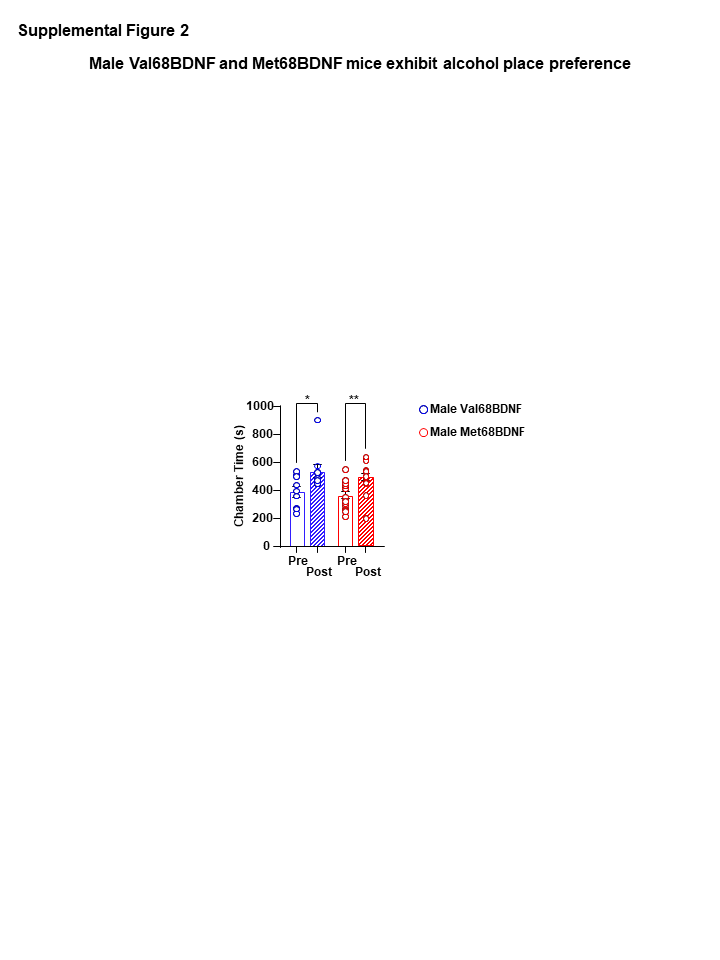

Supplement: Supplementary file 3 — Male Val68BDNF and Met68BDNF mice exhibit alcohol place preference Mice underwent an alcohol place preference paradigm in which they were first able to freely explore both chambers (pre-test). On alternating conditioning days, mice were placed in a saline- or alcohol-paired chamber after receiving an i.p. injection of saline or 2 g/kg of alcohol. During the post-test day, mice were once again allowed to freely explore the entire apparatus and time spend in each of the chambers were recorded and quantified. Male Val68BDNF (blue) and Met68BDNF (red) mice exhibit a significantly higher preference for the alcohol-paired chamber compared with the saline-paired chamber. All data are represented as mean ± SEM; * p < 0.05. Val68BDNF: n = 9 (2 were removed due to health issues), Met68BDNF: n = 17. (PNG 23 kb) [file 213_2022_6305_Fig7_ESM.png]

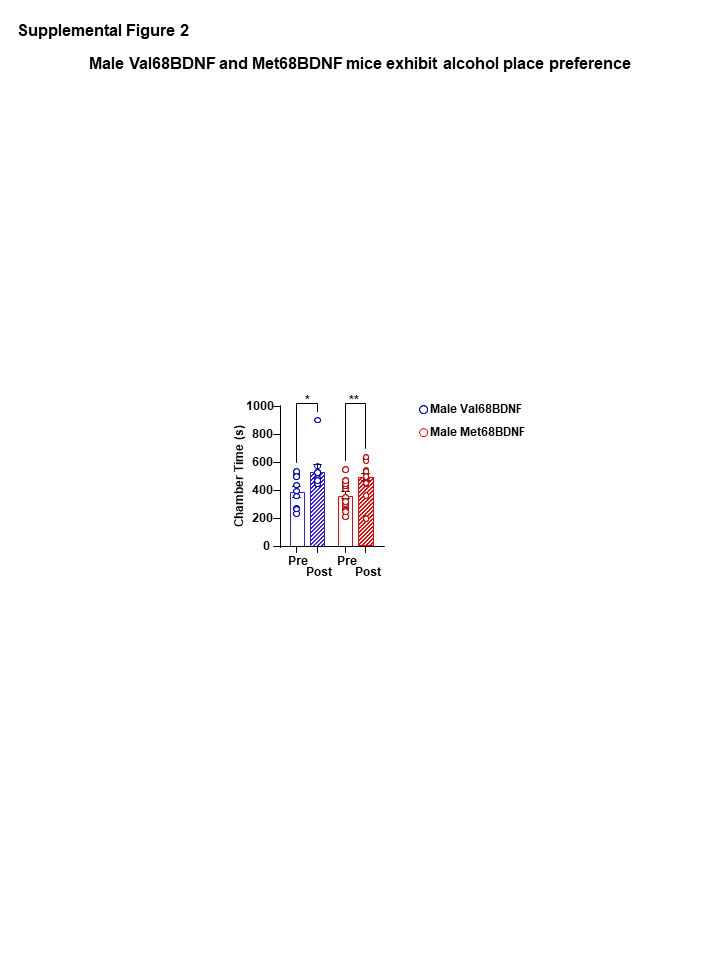

Supplement: Supplementary file 4 — High Resolution (TIF 55 kb) [file 213_2022_6305_MOESM2_ESM.tif]

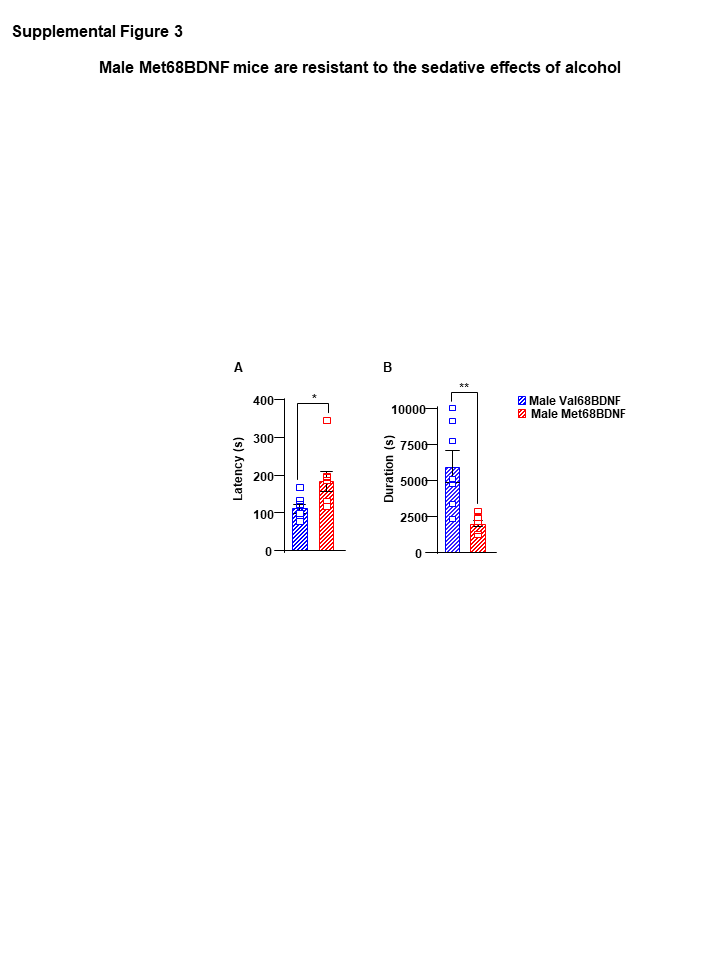

Supplement: Supplementary file 5 — Male Met68BDNF mice are resistant to the sedative effects of alcohol (A) Mice received 4 g/kg of alcohol and the time it took for sedation to set in, and the duration of the sedation were recorded. The latency between alcohol injection (4 g/kg) and the point at which mice do not right themselves after being placed on their backs was significantly greater in male Met68BDNF mice (red) than male Val68BDNF mice (blue). (B) The total duration of LORR for male Met68BDNF mice (red) was significantly shorter than it was for mice Val68BDNF mice (blue). Data are represented as mean ± SEM; * p < 0.05, ** p < 0.01. Val68BDNF: n = 7, Met68BDNF: n = 8.(PNG 20 kb) [file 213_2022_6305_Fig8_ESM.png]

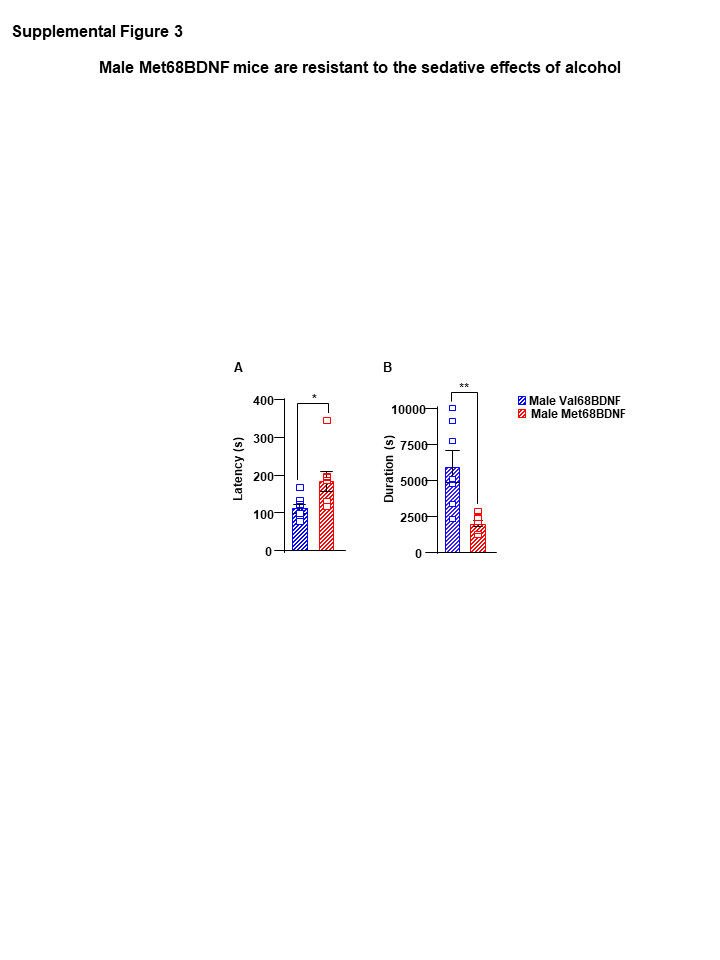

Supplement: Supplementary file 6 — High Resolution (TIF 54 kb) [file 213_2022_6305_MOESM3_ESM.tif]

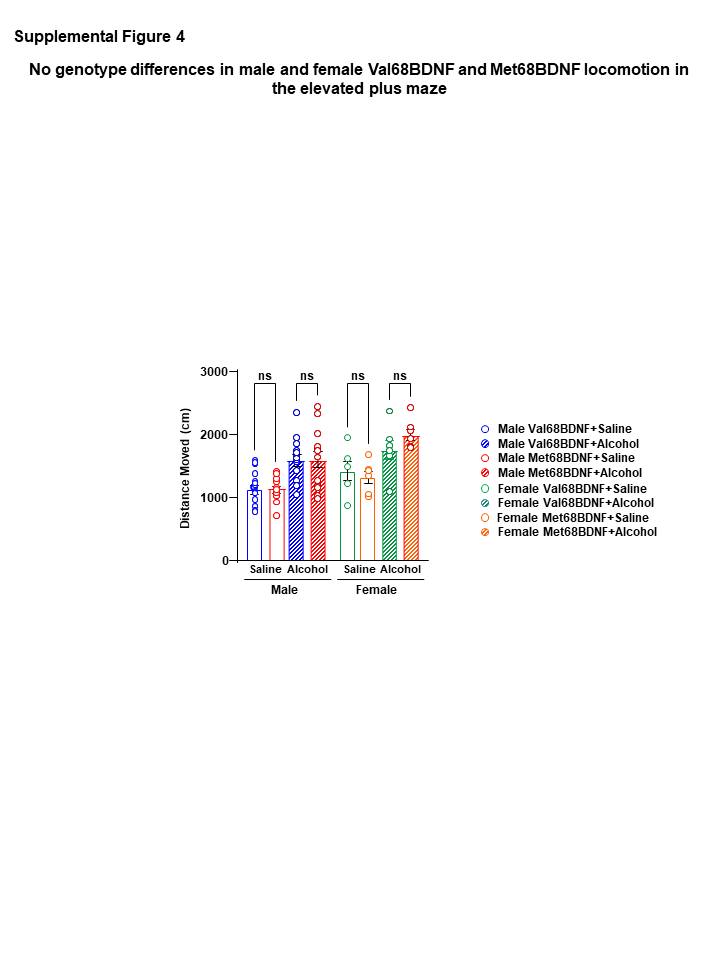

Supplement: Supplementary file 7 — No genotype differences in male and female Val68BDNF and Met68BDNF locomotion in the elevated plus maze Male Met68BDNF (red) mice travel the same distance on the elevated plus maze as male Val68BDNF (blue) mice following i.p. injection of saline (empty bars) or 1.25 g/kg of alcohol (hashed bars). Female Val68BDNF (green) and Met68BDNF (orange) mice also travel the same distance after i.p. injection of saline (empty bars) or 1.25 g/kg of alcohol (hatched bars). Data are represented as mean ± SEM. Male Val68BDNF + saline: n = 15, male Val68BDNF + alcohol: n = 14, male Met68BDNF + saline: n = 13, male Met68BDNF + alcohol: n = 14, female Val68BDNF + saline: n = 6, female Val68BDNF + alcohol: n = 7, female Met68BDNF + saline: n = 7, female Met68BDNF + alcohol: n = 7.(PNG 34 kb) [file 213_2022_6305_Fig9_ESM.png]

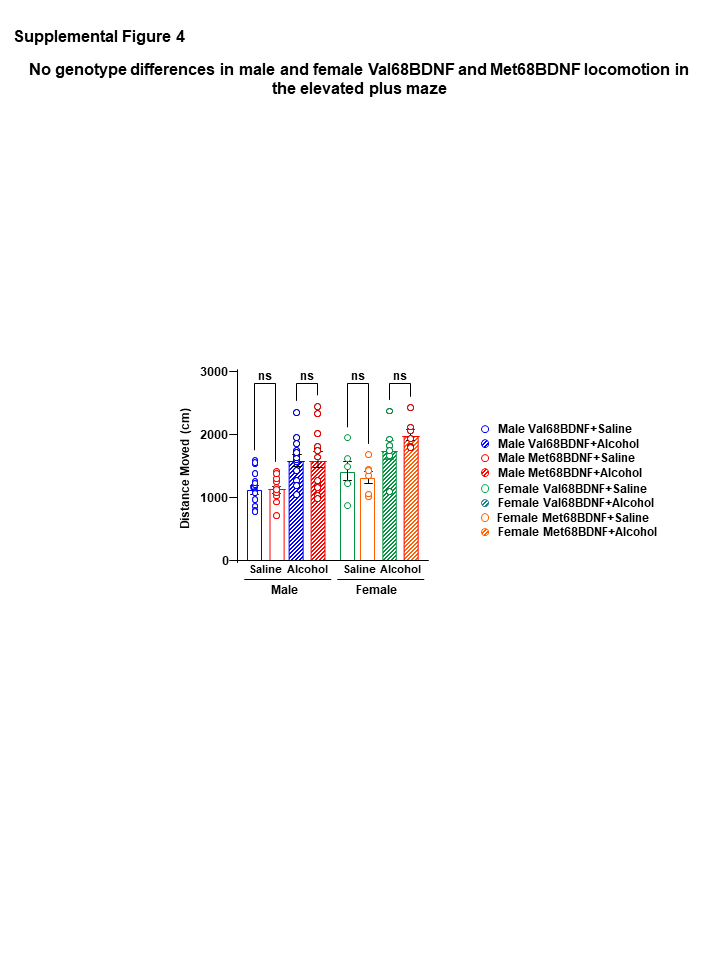

Supplement: Supplementary file 8 — High Resolution (TIF 84 kb) [file 213_2022_6305_MOESM4_ESM.tif]

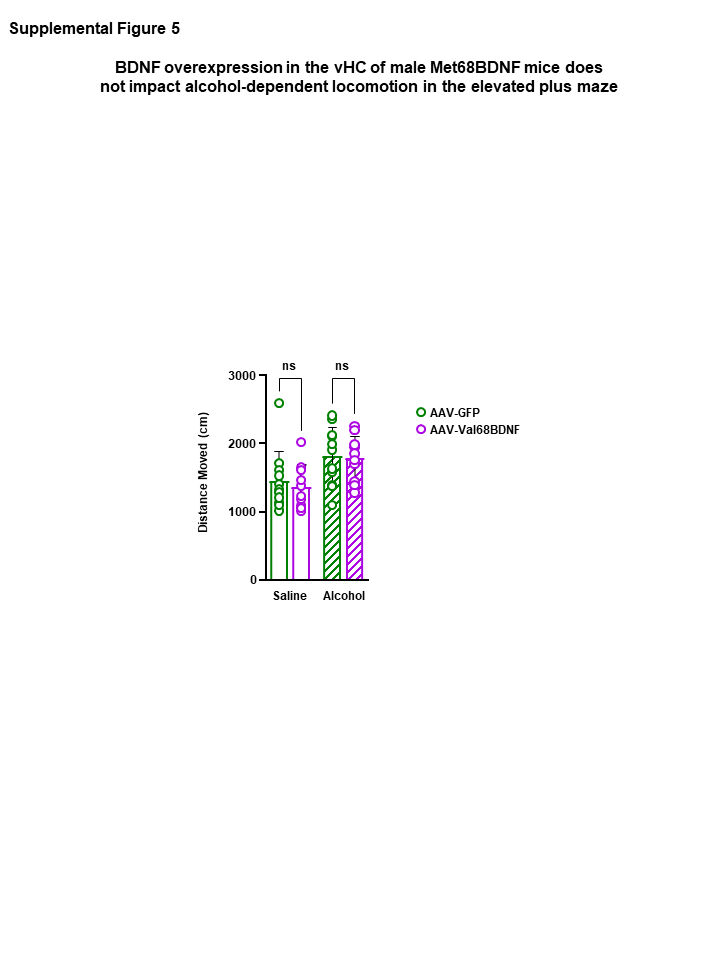

Supplement: Supplementary file 9 — BDNF overexpression in the vHC of male Met68BDNF mice does not impact alcohol-dependent locomotion in the elevated plus maze Male Met68BDNF mice that received AAV-Val68BDNF in the vHC (purple) move the same distance as male Met68BDNF mice that received AAV-GFP in the vHC (green) following i.p. injection of either saline (empty bars) or 1.25 g/kg of alcohol (hatched bars). Data represented as mean ± SEM. Male Met68BDNF + AAV-GFP: n = 11, Male Met68BDNF + AAV-Val68BDNF: n = 10. (PNG 28 kb) [file 213_2022_6305_Fig10_ESM.png]

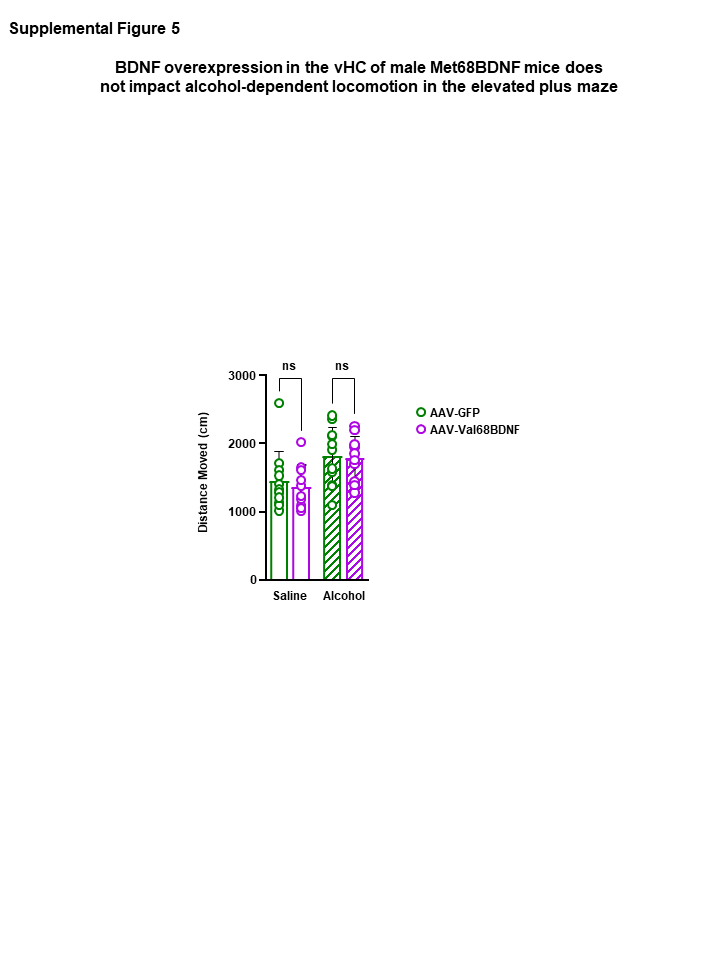

Supplement: Supplementary file 10 — High Resolution (TIF 66 kb) [file 213_2022_6305_MOESM5_ESM.tif]
